# Imaging-based clusters in current smokers of the COPD cohort associate with clinical characteristics: the SubPopulations and Intermediate Outcome Measures in COPD Study (SPIROMICS)

**DOI:** 10.1186/s12931-018-0888-7

**Published:** 2018-09-18

**Authors:** Babak Haghighi, Sanghun Choi, Jiwoong Choi, Eric A. Hoffman, Alejandro P. Comellas, John D. Newell, R. Graham Barr, Eugene Bleecker, Christopher B. Cooper, David Couper, Mei Lan Han, Nadia N. Hansel, Richard E. Kanner, Ella A. Kazerooni, Eric A. C. Kleerup, Fernando J. Martinez, Wanda O’Neal, Stephen I. Rennard, Prescott G. Woodruff, Ching-Long Lin

**Affiliations:** 1 Department of Mechanical and Industrial Engineering, University of Iowa, 2406 Seamans Center for the Engineering Art and Science, Iowa City, Iowa 52242 USA; 2 IIHR-Hydroscience & Engineering, University of Iowa, 2406 Seamans Center for the Engineering Art and Science, Iowa City, Iowa 52242 USA; 3 Department of Mechanical Engineering, Kyungpook National University, Daegu, Republic of Korea; 4 Department of Radiology, University of Iowa, Iowa City, Iowa USA; 5 Department of Epidemiology, Mailman School of Public Health, Columbia University Medical School, New York, NY USA; 6 Division of Genetics, Genomics and Precision Medicine, Department of Medicine, University of Arizona, Tucson, AZ USA; 7 Department of Physiology, UCLA, Los Angeles, CA USA; 8 Department of Biostatistics, University of North Carolina, Chapel Hill, NC USA; 9 Department of Internal Medicine, University of Michigan, Ann Arbor, MI USA; 10 School of Medicine, Johns Hopkins, Baltimore, MD USA; 11 School of Medicine, University of Utah, Salt Lake City, UT USA; 12 Department of Radiology, University of Michigan, Ann Arbor, MI USA; 13 Department of Medicine, UCLA, Los Angeles, CA USA; 14 Department of Medicine, Weill Cornell Medical Center, New York, NY USA; 15 School of Medicine, University of North Carolina, Chapel Hill, NC USA; 16 Department of Internal Medicine, University of Nebraska College of Medicine, NE, USA and Clinical Discovery Unit, AstraZeneca, Cambridge, UK; 17 Department of Medicine, University of California San Francisco, San Francisco, CA USA

**Keywords:** COPD, Current smokers, Emphysema, Functional small airway disease, Imaging-based cluster analysis

## Abstract

**Background:**

Classification of COPD is usually based on the severity of airflow, which may not sensitively differentiate subpopulations. Using a multiscale imaging-based cluster analysis (MICA), we aim to identify subpopulations for current smokers with COPD.

**Methods:**

Among the SPIROMICS subjects, we analyzed computed tomography images at total lung capacity (TLC) and residual volume (RV) of 284 current smokers. Functional variables were derived from registration of TLC and RV images, e.g. functional small airways disease (fSAD%). Structural variables were assessed at TLC images, e.g. emphysema and airway wall thickness and diameter. We employed an unsupervised method for clustering.

**Results:**

Four clusters were identified. Cluster 1 had relatively normal airway structures; Cluster 2 had an increase of fSAD% and wall thickness; Cluster 3 exhibited a further increase of fSAD% but a decrease of wall thickness and airway diameter; Cluster 4 had a significant increase of fSAD% and emphysema. Clinically, Cluster 1 showed normal FEV1/FVC and low exacerbations. Cluster 4 showed relatively low FEV1/FVC and high exacerbations. While Cluster 2 and Cluster 3 showed similar exacerbations, Cluster 2 had the highest BMI among all clusters.

**Conclusions:**

Association of imaging-based clusters with existing clinical metrics suggests the sensitivity of MICA in differentiating subpopulations.

**Electronic supplementary material:**

The online version of this article (10.1186/s12931-018-0888-7) contains supplementary material, which is available to authorized users.

## Background

Chronic obstructive pulmonary disease (COPD) is currently the third leading cause of death in the United States [1]. COPD is characterized by airflow limitation that is incompletely reversible [2], and thus it is identified by the ratio of forced expiratory volume in 1 s over forced vital capacity (FEV1/FVC) at post bronchodilator. The severity is further distinguished by FEV1% predicted values by COPD guidelines [3]. The ratio of FEV1/FVC is used as an indicator to identify COPD patients in diagnosis of the disease [3], but it may not be sensitive enough to differentiate heterogeneous alterations characterized by multiple pathologies [4]. In contrast, quantitative computed tomography (QCT) can distinguish emphysema-predominant and airway-predominant diseases [5] and help link structural and functional variables [6, 7]. Individual imaging-based metrics have been derived from both CT and MRI studies of the lungs in both COPD and asthma [8]. With recent advances in unsupervised clustering of subject populations [9–11], there is an increased effort to employ these methods for grouping sub-populations of subjects within both the asthma [12] and COPD communities [13–17].

With the introduction of novel structural and functional imaging-based metrics [6] and corrections for inter-site and inter-subject variabilities [18], Choi et al. [7] recently integrated all of the imaging-based metrics measured at multi-scales to derive imaging-based clusters of subjects from an asthma population. These clusters were significantly associated with clinical characteristics. In the present work, we utilize the same approach, but with an expanded set of variables that include an image matching-based quantification of emphysema and functional small airways disease to derive imaging-based clusters in a COPD population with meaningful associations to clinical characteristics. For this purpose we investigated a subject population from within the Subpopulations and Intermediate Outcome Measures in COPD Study (SPIROMICS) [19] which was initiated to provide robust criteria for sub-classifying COPD participants and further identify biomarkers and phenotypes for efficient conduct of treatment trials.

## Methods

### Human data and QCT imaging

From the first 1000 subjects recruited into SPIROMICS [19] we performed image matching and identified 700 subjects in whom total lung capacity (TLC) to residual volume (RV) matches were successful. From these subjects with matching data we chose to study current smokers falling within strata 2–4 [19] (*N* = 284) as well as healthy non-smokers (*N* = 130). SPIROMICS categorized subjects into four strata 1–4. The healthy non-smokers (stratum 1) were defined as FEV1/FVC > 0.7 with smoking status (pack-year) < 1. Smokers with (pack-year) > 20 and FEV1/FVC > 0.7 were grouped in stratum 2. Also smokers in strata 3 and 4 had FEV1/FVC < 0.7; those with FEV1 > 50% were grouped in stratum 3 whereas those with FEV1 < 50% were in stratum 4 [19]. The demographics of these populations are summarized in Table 1. The current smokers were employed to derive imaging-based COPD clusters and individual metrics were compared with the non-smoking healthy controls. We initially performed cluster analysis [20] including both former and current smokers, which resulted in less statistically stable clusters based on the Jaccard index [21] (90% and 70% for current and both former and current smokers, respectively). This suggested that smoking status introduced confounding variables, interfering with many metrics such as the emphysema index which is shifted by the effect of inflammation (associated with smoking status) on regional lung density [22].

**Table 1:** Demography, baseline (Pre-bronchodilator) and maximal (Post-bronchodilator) pulmonary function tests for 130 Stratum 1 (healthy), 114 Stratum 2, 131 Stratum 3 and 39 Stratum 4 subjects

	Stratum 1 (Healthy)	Stratum 2	Stratum 3	Stratum 4	*P* value
	*N* = 130	*N* = 114	*N* = 131	*N* = 39	
Demography					
Age, yrs	47.8 (16.9)	53.7 (8.1)	62 (8.1)	62.6 (7.7)	< 0.0001
BMI, kg/m^2^	27.4 (5.5)	28.4 (5.3)	26.3 (4.8)	23.8 (4.9)	< 0.0001
Gender, (Male/Female %)	41.5/58.5	49.1/50.9	61.8/38.2	69.2/30.8	0.039
Race, Caucasian/ African American/ Other (%)	71.5/16.2/12.3	45.6/48.2/6.1	77.1/19.1/3.8	71.8/20.5/7.7	< 0.0001
Baseline lung function^a^					
FEV_1_% predicted	100 (13)	93 (14)	64 (18)	34 (7)	< 0.0001
FVC % predicted	99 (11)	98 (14)	87 (19)	67 (16)	< 0.0001
FEV_1_/FVC × 100	80 (7)	75 (6)	56 (8)	40 (11)	< 0.0001
Maximal lung function^b^					
FEV_1_% predicted	102 (11)	99 (14)	73 (16)	40 (7)	< 0.0001
FVC % predicted	99 (10)	100 (13)	96 (18)	78 (17)	< 0.0001
FEV_1_/FVC × 100	82 (6)	78 (5)	58 (8)	41 (11)	< 0.0001

Values expressed as mean (SD) or number (%). Kruskal-Wallis and chi-square tests were performed for continuous and categorical variables. ^a^Baseline (Prebronchodilator) values with greater than six hours withhold of bronchodilators. ^b^Maximal (Postbronchodilator) values after six to eight puffs of albuterol. Maximal lung function for 25 healthy subjects were not available

### Multiscale imaging-based variables

Volumetric CT imaging was carried out during coached breath holds at TLC and RV [23], and image analysis was carried out with use of the Apollo software (VIDA Diagnostics, Coralville, Iowa).

Sixty nine post-processed imaging-based variables were employed at both segmental and lobar levels, which is an expanded set of existing 57 variables used for asthma cluster analysis [7] utilizing our multiscale imaging-based clustering approach (MICA). The four structural variables at the pre-segmental and segmental levels were extracted from ten local regions to reflect the regional characteristics [6]. These structural variables included bifurcation angle (*θ*), airway circularity (*Cr*), wall thickness (WT) and hydraulic diameter (*D*_*h*_), where each variable indicated alteration of airway geometry, alteration of luminal shape, wall thickening and luminal narrowing, respectively. The dimensions of WT and *D*_*h*_ were normalized by predicted trachea WT and *D*_*h*_ from healthy controls denoted by WT* and *D*_h_* [6]. The normalization was used for eliminating inter-subject variability due to sex age and height.

Employing a mass-preserving image registration technique [24, 25], lobar/global functional variables were further derived to describe the alterations of lung deformation between inspiration and expiration. The variables at lobar levels included fractional air volume change (ΔV_air_^F^), the determinant of Jacobian matrix (Jacobian) [26] and anisotropic deformation index (ADI) [26, 27], indicating regional contribution of ventilation (lobar fraction of air volume change between TLC and RV), regional volume change, and the degree of preferential deformation, respectively. In this study, we also employed three new variables; fraction-based small airways disease (fSAD%) to characterize small airway, fraction-based emphysema (Emph%) for emphysematous diseases as well as tissue fraction at TLC (β_tissue_). Emph % and fSAD% were defined based upon a variation of the image-matching-based parametric response map used by Galban et al. [28]. In our implementation, Emph% (98.5% air-fraction as the threshold) and fSAD% (90% air-fraction as the threshold) were used instead of using the density threshold identifying voxels < − 950 HU, to account for scanner variability [18]. β_tissue_ indicates the portion of tissue volume in each voxel to assess a possible alteration of local tissue. Also, related global (whole lung) variables were included; fSAD% (Total) and Emph% (Total), apical-basal distance over ventral-dorsal distance at TLC (lung shape), the ratio of air-volume changes in upper lobes to those in middle and lower lobes between TLC and RV (U/(M + L)|v), Jacobian (Total) and ADI (Total) [26]. Therefore, we obtained 32 local structural as well as 30 lobar and 7 global variables, giving 69 imaging-based variables. These comprehensive imaging variables were then used for a cluster analysis. Full names of each variable are described in *Abbreviations used* section.

### Clustering and statistical analysis

**Fig. 1:**
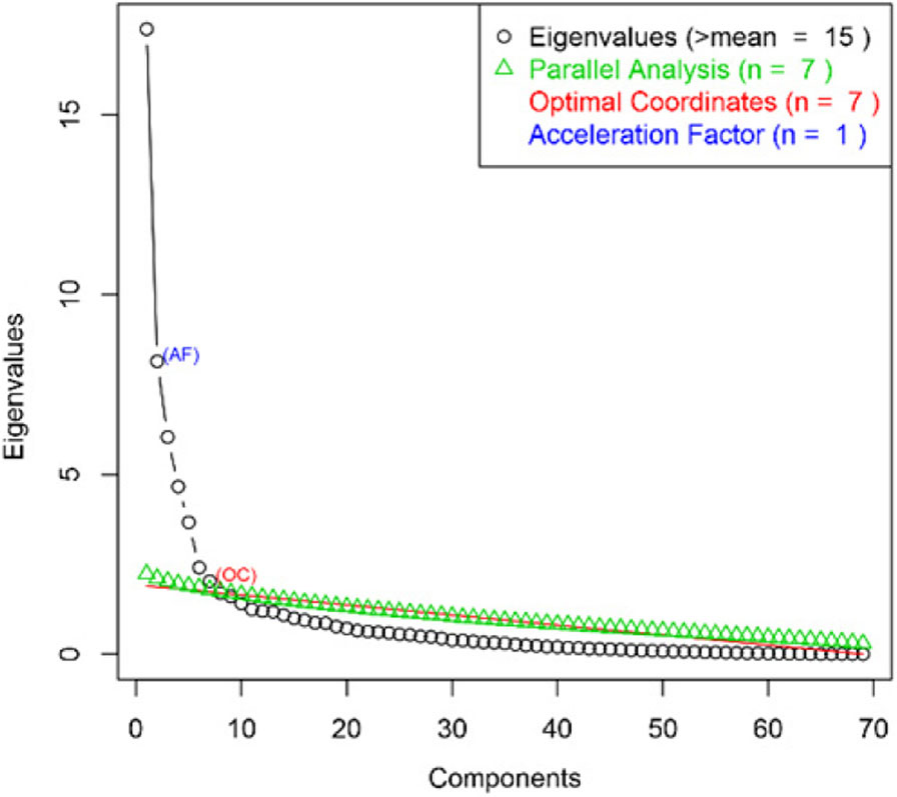
A scree plot for determining the optimal number of principal components

**Fig. 2:**
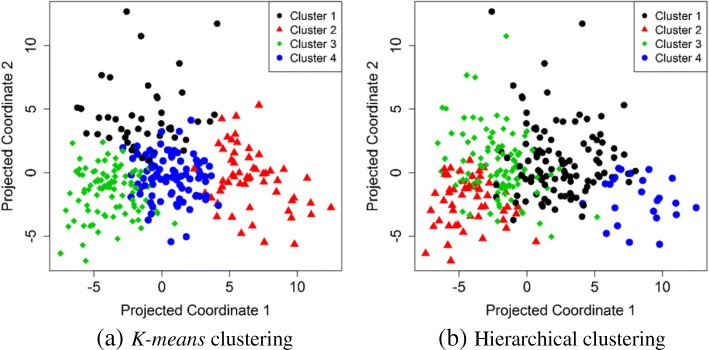
**a** Clustering membership of *K-mean*s clustering on 2-D projected coordinates; (**b**) Clustering membership of Hierarchical clustering on 2-D projected coordinates

We compared three general clustering methods including *K-means*, Hierarchical [29] and Gaussian finite mixture model-based [30] fed by principal components [31, 32]. Three methods (Kaiser/Harris, Cattel Scree Test and Parallel Analysis [33]) were performed to retain an optimal number of principal components (Fig. 1). Internal properties of clusters (Connectivity, Dunn index and Silhouette indices) were used in order to find the best clustering method. The *K-means* clustering method showed more stability and an optimal number of clusters fitted for the structure of the imaging data (Additional file 1). The results of clustering for K-means and hierarchical are shown in Fig. 2. K-means clustering with could achieve more clear separation of the cluster membership compared to hierarchical clustering.

Next, we performed association tests of imaging-based clusters with demographic and clinical variables to investigate the clinical relevance of current clusters. The data analysis was performed by *R* software (version 3.1.1). Kruskal-Wallis and chi-square tests were performed to compare differences of continuous and categorical variables, respectively. *P* = 0.05 was taken as the significant level in all tests. The validation of the cluster analysis was assessed by dividing the data set into training and validation sets (see the Additional file 1).

## Results

### Four clusters and imaging-based characteristics

**Fig. 3:**
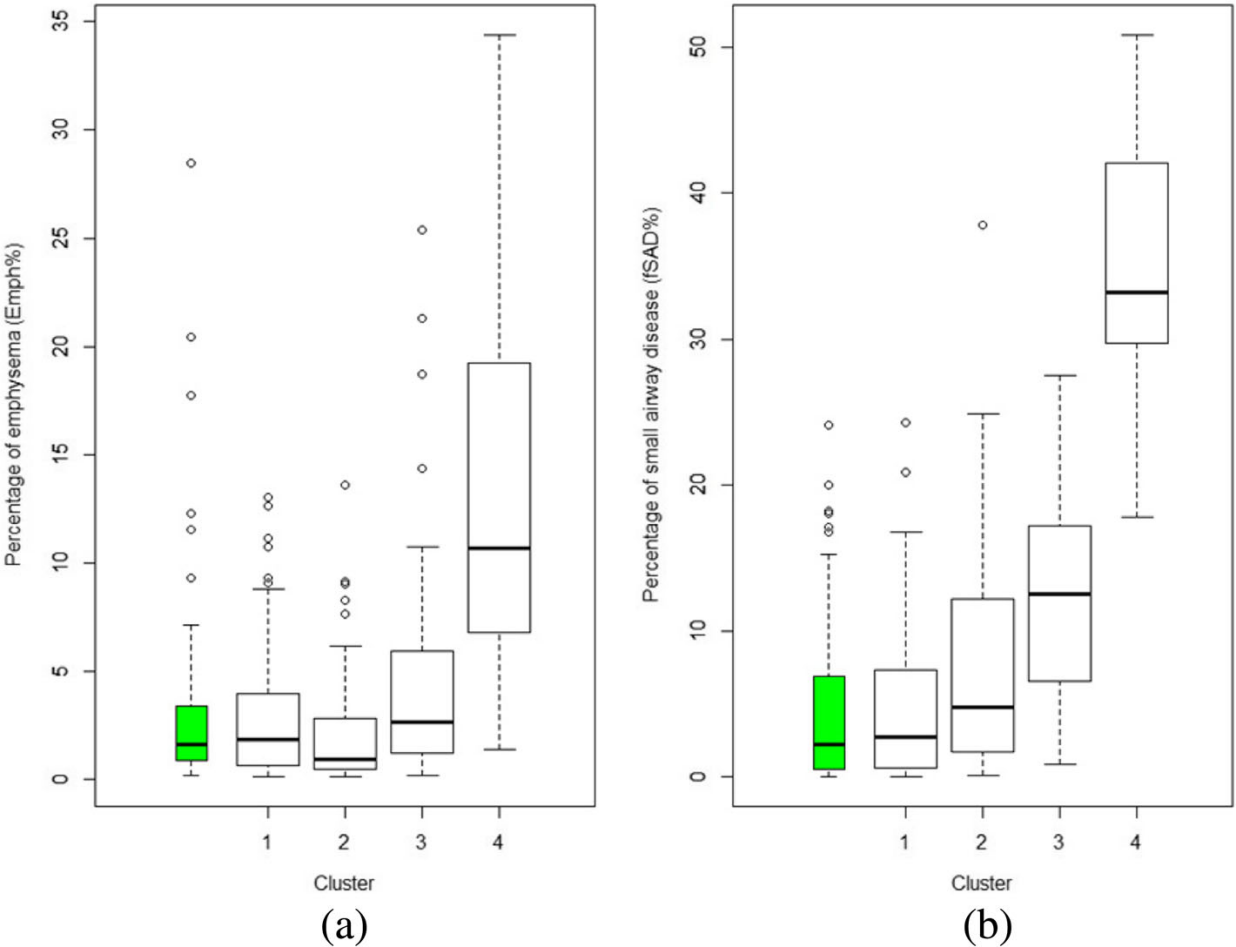
**a** Percentage of emphysema (Emph%) for four clusters and the healthy control group (green). † *P* > 0.05 between clusters 1, 2, 3 and the healthy group. *P* < 0.05 between Cluster 4 and other groups for all pairwise comparisons (**b**) Percentage of small airway disease (fSAD%) for four clusters and the healthy control group (green). ‡ *P* < 0.05 for comparisons between four clusters 2, 3, 4 and the healthy group for all pairwise comparison. *P* > 0.05 for between Cluster 1 and the healthy group

**Fig. 4:**
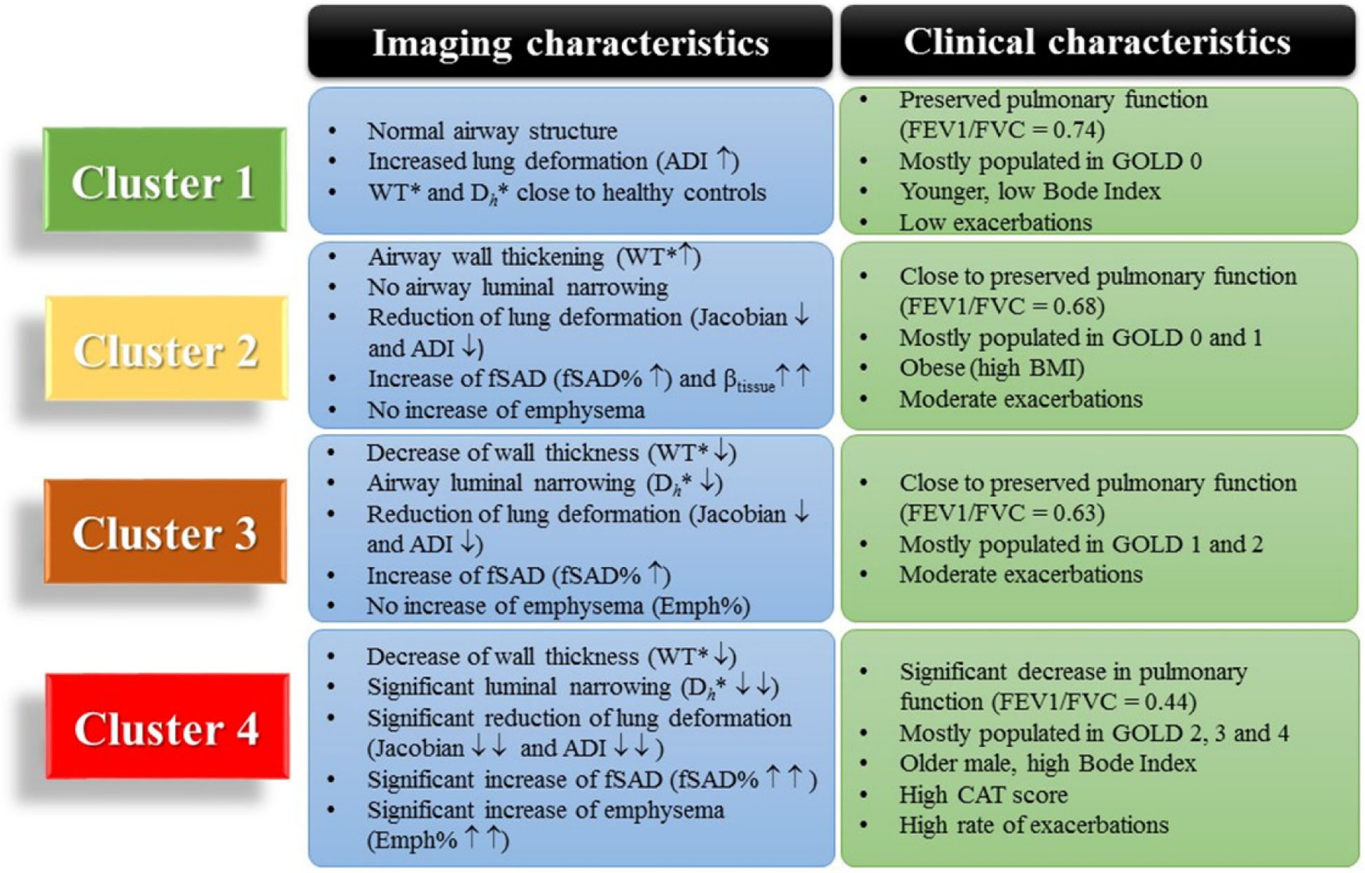
A summary of imaging and clinical variables for four clusters

**Fig. 5:**
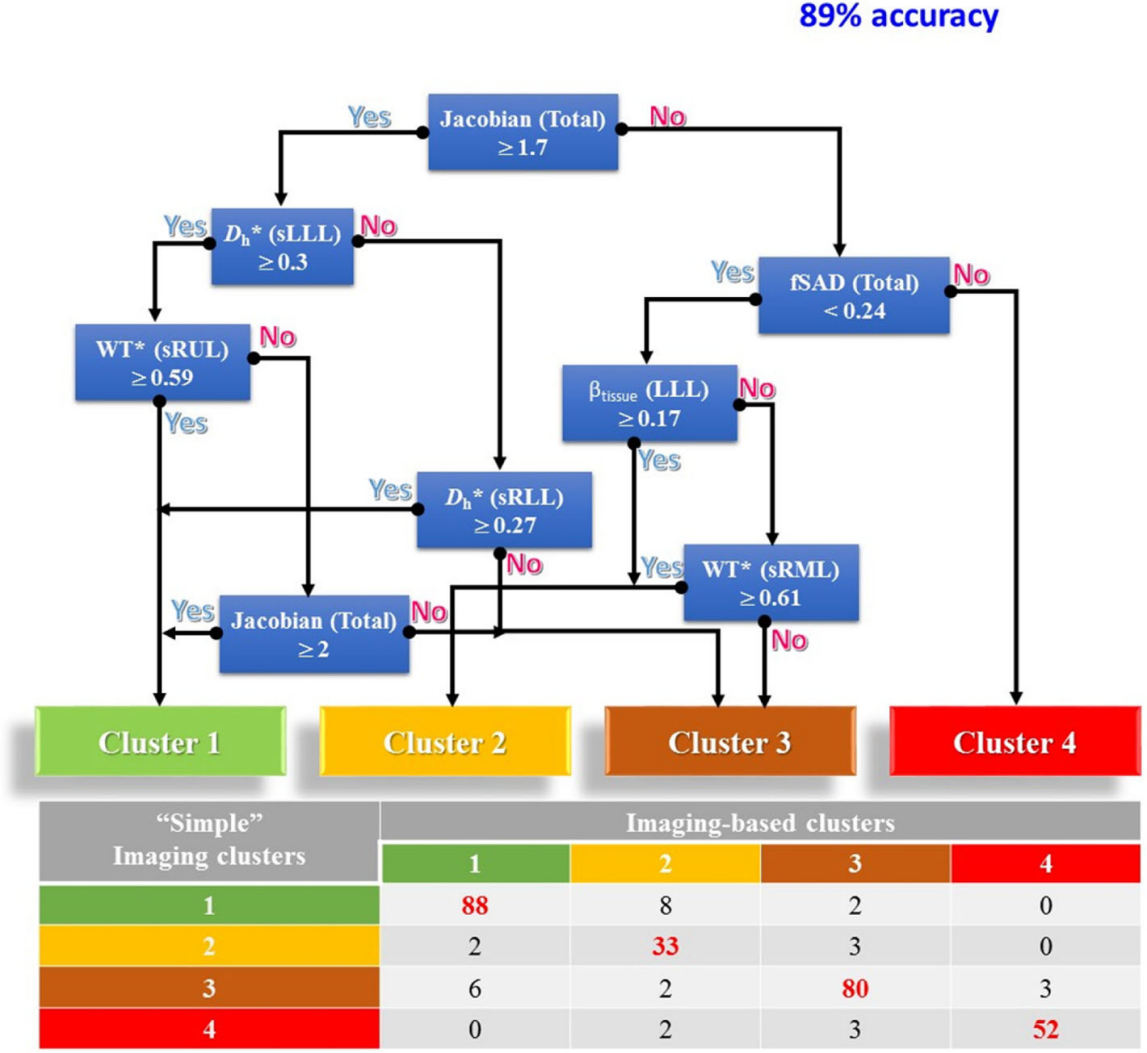
Predicting imaged-based cluster using only 7 important variables with a classification tree (“simple” imaging-based clustering). Variables are Jacobian (Total), *D*_h_* (sLLL), *D*_h_* (sRLL), WT* (sRUL), WT* (sRML), β_tissue_ (LLL) and fSAD% (Total) with 89% accuracy compared with “original” imaging-based clusters using 69 variables

The K-means clustering method produced four unique clusters, containing 96, 45, 88 and 55 subjects respectively (Table 2). Figure 3 shows the percentages of emphysema and small airway disease (Emph% and fSAD%) for the different clusters and the healthy group. Figure 4 summarizes the imaging-based characteristics of the four clusters. The major variables which best describe the four clusters were selected with a stepwise forward variable selection technique using Wilk’s *λ* criterion [34]. Ten major variables with higher Wilk’s *λ* values are presented to explain structural and functional alterations associated with each cluster (Table 2). We then performed a decision tree analysis to construct a simple predictive model (Fig. 5). The model comprising 7 discriminant variables achieved 89% accuracy in classification. These variables were Jacobian (Total), *D*_h_* (sLLL), *D*_h_* (sRLL), WT* (sRUL), WT* (sRML), β_tissue_ (LLL) and fSAD% (Total).

**Table 2:** Major structural and functional imaging-based variables in four imaging-based clusters and heathy subjects

Variable	Region	Wilk’s λ value	Cluster 1 (*N* = 96)	Cluster 2 (*N* = 45)	Cluster 3 (*N* = 88)	Cluster 4 (*N* = 55)	*P* value	Healthy subjects (*N* = 130)
fSAD%	Total	0.28	4.6 (5.4)	8.4 (8.2)	12.3 (6.9)	34.9 (7.9)	< 0.0001	4.4 (5.2)
Jacobian	Total	0.145	2.09 (0.266)	1.496 (0.218)	1.671 (0.168)	1.353 (0.136)	< 0.0001	2.082 (0.41)
β_tissue_	Total	0.107	0.127 (0.02)	0.162 (0.031)	0.117 (0.017)	0.095 (0.019)	< 0.0001	0.119 (0.027)
WT*	sRML	0.086	0.599 (0.036)	0.615 (0.047)	0.557 (0.035)	0.563 (0.043)	< 0.0001	0.588 (0.047)
ADI	RUL	0.072	0.406 (0.078)	0.314 (0.101)	0.309 (0.079)	0.22 (0.074)	< 0.0001	0.35 (0.093)
*D*_h_*	sLLL	0.064	0.349 (0.034)	0.322 (0.048)	0.307 (0.036)	0.289 (0.04)	< 0.0005	0.339 (0.041)
Emph%	Total	0.058	2.8 (3)	2.4 (3)	4.2 (4.5)	13.5 (8.7)	< 0.0001	2.8 (3.8)
ADI	Total	0.054	0.467 (0.066)	0.332 (0.086)	0.378 (0.07)	0.269 (0.073)	< 0.0001	0.429 (0.101)
Δ*V*_air_^F^	LLL	0.051	0.245 (0.031)	0.207 (0.062)	0.254 (0.041)	0.273 (0.045)	< 0.0001	0.263 (0.037)
Cr	LMB	0.049	0.976 (0.009)	0.965 (0.016)	0.973 (0.012)	0.962 (0.015)	< 0.0001	0.977 (0.011)

Values expressed as mean (SD). The major imaging-based variables were selected by Wilk’s *λ* value of a stepwise forward variable selection method. Analysis of variance (ANOVA) tests were performed to attain *P* values. WT and D_h_ were normalized with their predicted trachea values from healthy controls denoted by WT* and D_h_*. Full names of each variable or region were described in *Abbreviations used*

### Associations with demography and PFT

Association of clusters with demography and PFTs are tabulated in Table 3. Cluster 1 with normal airway structures was mostly populated by GOLD 0 and stratum 2 subjects who were relatively younger and presented with a lower BODE index compared to other clusters (*P* < 0.05). Unlike Cluster 1, Cluster 4 was mostly populated by GOLD 2, 3, 4 and strata 3, 4, respectively with relatively older subjects. Cluster 1 included most subjects from group A and B from ABCD assessment while subjects in Cluster 4 shifted towards group D. Cluster 2 was associated with relatively higher BMI subjects. Also Cluster 3 was associated with subjects who exhibited a relatively low BODE index. Cluster 4 subjects showed higher BODE index and were relatively older males.

**Table 3:** Demography, baseline (Pre-bronchodilator) and maximal (Post-bronchodilator) PFTs, in four imaging-based clusters

	Cluster 1	Cluster 2	Cluster 3	Cluster 4	*P* value
	*N* = 96	*N* = 45	*N* = 88	*N* = 55	
Demography					
GOLD stages (%) (0/1/2/3/4)	68/22/10/0/0	47/11/33/9/0	27/15/51/7/0	4/2/40/47/7	< 0.0001
ABCD assessment (%) (A/B/C/D)	37/47/1/14	25/52/0/22	35/49/0/16	10/51/0/39	= 0.0005
Strata (%) (2/3/4)	68/32/0	51/40/9	27/66/7	4/44/52	< 0.0001
Gender (Female %)	44	49	44	31	0.26
Race (White/Black/Others)	60/32/7	36/58/7	72/26/2	80/15/5	0.0001
Age (yrs.)	54.44 (8.01)	56.76 (8.51)	61.01 (8.24)	64.47 (8.14)	< 0.0001
BMI (kg/m2)	27.63 (4.7)	31.1 (5.04)	25.58 (4.76)	23.65 (4.26)	< 0.0001
BODE index	0.48 (0.9)	1.4 (1.9)	0.98 (1.1)	2.94 (1.7)	< 0.0001
Pre-bronchodilator values^a^					
FEV1% predicted	0.91 (0.17)	0.73 (0.22)	0.68 (0.2)	0.42 (0.17)	< 0.0001
FVC % predicted	1.01 (0.15)	0.86 (0.17)	0.86 (0.18)	0.74 (0.16)	< 0.0001
FEV1/FVC	0.71 (0.09)	0.66 (0.14)	0.61 (0.1)	0.43 (0.11)	< 0.0001
Post-bronchodilator values^b^					
FEV1% predicted	0.97 (0.16)	0.8 (0.2)	0.76 (0.18)	0.49 (0.17)	< 0.0001
FVC % predicted	1.04 (0.15)	0.92 (0.16)	0.93 (0.17)	0.85 (0.16)	< 0.0001
FEV1/FVC	0.74 (0.09)	0.68 (0.13)	0.63 (0.11)	0.44 (0.12)	< 0.0001

Data presented as number (%) or mean (SD). ANOVA and chi-square tests were performed for continuous and categorical variables, respectively. ^a^Pre-bronchodilator values. ^b^Post-bronchodilator values after six to eight puffs of albuterol. Full names of each variable were described in Abbreviations used. BODE indexes for 8 subjects were not available

Both pre-bronchodilator and post-bronchodilation PFT-derived lung function values are tabulated in Table 3. FEV1/FVC showed a consistent, decreasing pattern from Cluster 1 to Cluster 4. Subjects in Cluster 4 demonstrated significant decreases in FEV1/FVC both pre-and post- bronchodilation, while Cluster 1 showed a mean FEV1/FVC of 0.74 that is above the normal cut off value of 0.7. A similar decreasing pattern from Cluster 1 to Cluster 4 was found for FEV1 and FVC % predicted values, with the highest and lowest values associated with Cluster 1 and Cluster 4, respectively.

### Associations with symptoms and disease histories

Symptoms and disease histories were collected from the SPIROMICS [19, 23] data set and are summarized in Table 4. Cluster 4 showed a higher history of chronic bronchitis, emphysema, wheezing and whistling compared to Clusters 1, 2, and 3. The prevalence of symptoms in Clusters 1, 2 and 3 was less likely than Cluster 4. Cluster 2 showed an increased history of sleep apnea diagnosed at baseline compared to other clusters. Cluster 4 had higher smoking pack-years at baseline (*P* < 0.05) compared to the other clusters.

**Table 4:** Associations with Symptoms and Disease Histories

	Cluster 1	Cluster 2	Cluster 3	Cluster 4	*P* value
	*N* = 96	*N* = 45	*N* = 88	*N* = 55	
Symptoms and disease History					
History of pulmonary/vascular condition (%)	19	13	24	23	0.53
Smoking pack-years at baseline	41.79 (22.05)	42.89 (18.7)	47.06 (19.36)	54.95 (21.03)	0.0016
Chronic Bronchitis (%)	16	20	20	39	0.016
Emphysema (%)	17	20	25	55	< 0.0001
COPD diagnosed at baseline (%)	29	51	58	83	< 0.0001
Chronic bronchitis diagnosed at baseline (%)	21	33	28	41	0.079
Asthma (%)	20	36	22	21	0.19
Wheezing and whistling in chest (%)	60	62	66	89	0.002
Wheezing age (yrs.) (%)	60	86	84	92	0.0003
Sleep Apnea at baseline (%)	6	21	7	6	0.02
Shortness of breath during sleep (%)	18	29	14	29	0.06
Coronary artery disease	2	4	9	6	0.19
Diabetes (%)	6	18	9	9	0.2
Heart attack (%)	3	4	6	2	0.65
Congestive heart failure (%)	3	0	1	0	0.33
Genetic effect					
Father had COPD (%)	19	13	23	22	0.61
Mother had COPD (%)	17	13	19	9	0.39

### CAT score, activity limitation and exacerbation histories

Blood biomarkers, baseline CAT score, exacerbation histories as well as activity limitation (6-min walk) are tabulated in Table 5. While clusters did not show significant difference in blood biomarkers, there was a significant difference for CAT score between clusters (*P* < 0.05). The CAT score for all clusters were more than 10, suggesting respiratory symptoms (symptomatic) in even the cluster 1 subjects, in agreement with the findings of Woodruff et al. [35]. While Clusters 1, 2 and 3 showed relatively similar CAT scores, Cluster 4 showed a higher CAT score than other clusters. Severe (since entering the study), total (since entering the study), and total (at baseline) exacerbations showed significant differences between clusters with Cluster 4 having the most severe exacerbations. There was no significant difference in the number of exacerbations between Clusters 1, 2 and 3. Also subjects in Clusters 2 and 4 were more likely to have activity limitations, as their 6-min walk distance and oxygen desaturation were lower than other Clusters.

**Table 5:** Characteristics of biomarkers in four imaging-based clusters

	Cluster 1	Cluster 2	Cluster 3	Cluster 4	*P* value
	*N* = 92	*N* = 45	*N* = 87	*N* = 55	
Blood/serum biomarkers					
RBC distribution width (%)	14.25 (1.17)	14.04 (0.87)	13.63 (0.84)	14.13 (1.19)	0.0016
Total WBC count (N/μl)	7153.15 (2290.62)	7352.89 (2527.31)	7109.77 (1954.51)	7073.09 (2122.85)	0.924
Neutrophils% (%)	58.06 (9.52)	58.24 (11.78)	58.06 (9.65)	61.14 (10.22)	0.26
Lymphocyte% (%)	31.35 (8.09)	31.39 (10.45)	30.81 (8.58)	27.74 (9.53)	0.088
Monocyte% (%)	7.3 (2.5)	7.46 (2.12)	7.67 (2.18)	7.85 (2.2)	0.512
Eosinophils% (%)	2.63 (1.64)	2.29 (1.74)	2.72 (1.75)	2.61 (1.9)	0.6
Basophils% (%)	0.71 (0.61)	0.52 (0.36)	0.62 (0.54)	0.68 (0.64)	0.278
Baseline CAT score^a^					
	13.17 (7.95)	16.45 (9.54)	13.78 (7.86)	20.06 (7.86)	< 0.0001
Exacerbations					
Severe^b^	0.2 (0.6)	0.44 (1.62)	0.31 (0.82)	1.25 (2.27)	< 0.0001
Total^c^	0.49 (1.19)	1.09 (3.39)	0.92 (2.14)	2.09 (2.91)	< 0.0001
Total at baseline^d^	0.25 (0.68)	0.58 (1.39)	0.22 (0.63)	0.62 (0.99)	0.011
Activity limitation					
6-min walk distance (m)	445.66 (91.31)	386.64 (136.27)	420.38 (71.19)	385.16 (94.09)	0.0003
Oxygen desaturation with 6-min walk (%)	14	36	14	41	< 0.0001

Biomarkers data for 5 subjects were not available. Kruskal-Wallis and chi-square tests were performed for continuous and categorical variables, respectively. ^a^CAT score range from 0 to 40, with higher scores indicating greater severity symptoms. ^b^Total count of exacerbations requiring ED visit or hospitalization since entering the study. ^c^Total count of exacerbations since entering the study. ^d^Total Exacerbations for baseline

### Cluster characteristics

#### Cluster 1: Relatively resistant smokers with preserved pulmonary function

Cluster 1 had increased smoking pack-years (41.79 ± 22.05) with no or minimal airway obstruction (FEV1/FVC = 0.74). Cluster 1 was mostly populated by GOLD stage 1 (66%) with low emphysema and low fSAD%. Cluster 1 showed that structural variables including WT*, *D*_h_* and *Cr* are very close to those of healthy controls. The CAT score, BODE index and severe exacerbation history of this cluster were relatively low compared to other clusters. Cluster 1 can be considered to be relatively resistant smokers with preserved pulmonary function.

#### Cluster 2: Airway-wall-thickening fSAD-dominant subjects with obesity and activity limitation

Cluster 2 had increased smoking pack-years (42.89 ± 18.7) and a FEV1/FVC relatively close to the lower limit of normal, 0.7. This cluster had the highest BMI among all clusters and a higher BODE index than Clusters 1 and 3. Cluster 2 exhibited a decrease of D_h_* and *Cr* compared to Cluster 1 and had the highest WT* and β_tissue_ and the lowest Jacobian among all clusters. Cluster 2 also showed an increase of fSAD%, but with Emph% close to that of Cluster 1. Cluster 2 showed no significant difference in the number of exacerbations or CAT score. Cluster 2 had decreased 6-min walk distance and oxygen desaturation, similar to Cluster 4 but decreased compared to Cluster 1, (*P* < 0.05). Thus, Cluster 2 can be classified as thickened airway wall, narrowed airway lumen and fSAD-dominant subjects with associated obesity and activity limitations.

#### Cluster 3: Airway-wall-thinning fSAD-dominant subjects

Compared to Clusters 1 and 2, Cluster 3 with smoking pack-years (47.06 ± 19.39, *P* > 0.05) showed a continued increase of fSAD% (*P* < 0.05) with similar Emph% (*P >* 0.05). D_h_* showed significant decrease as compared with Cluster 1, but not significant difference from that of Cluster 2. Also WT* decreased compared to Clusters 1 and 2 (*P* < 0.05). FEV1/FVC (=0.63) for Cluster 3 remained close to the normal range with no significant difference for the three categories of exacerbation (severe, total and total at baseline) between Clusters 1, 2 and 3. Cluster 3 had 58% of subjects in GOLD stages 2–4 and had a CAT score close to Clusters 1 and 2. While Cluster 3 did not show significant differences in 6-min walk distance or oxygen desaturation compared to Cluster 2, its oxygen desaturation decreased comparable to that of Cluster 2 (*P* < 0.05). Cluster 3 can be categorized as fSAD-dominant subjects with luminal narrowing and decreased wall thickness.

#### Cluster 4: Severe emphysema-fSAD-mixed subjects with severe airway luminal narrowing and wall thinning

Cluster 4 had significantly greater smoking pack-years (54.95 ± 21.03) compared to other clusters. It had a higher CAT score along with more exacerbations and greater activity limitations compared to the other clusters. Cluster 4 also showed significant elevation of emphysema and small airways disease (fSAD%↑↑ and Emph%↑↑), significant decreases in lung deformation (Jacobian↓↓ and ADI↓↓) and significant airway luminal narrowing (*D*_h_*↓↓, *P* < 0.05) compared to Clusters 1, 2, and 3. Cluster 4 had significant decreases in airway wall thickness (WT*↓↓, *P* < 0.05) compared to Clusters 1 and 2. Cluster 4 also had a much lower FEV1/FVC for both baseline function and maximal post-bronchodilator lung function compared to the other Clusters. Cluster 4 had a higher BODE index compared to the other clusters. Lymphocyte% decreased in Cluster 4 and reached near statistical significance (*P* = 0.08). Therefore, Cluster 4 subjects can be classified as severe mixed emphysema-fSAD with severe luminal narrowing, decreased wall-thickness and lung function.

## Discussion

In the present study, we applied MICA [7], which utilized an expanded set of 69 QCT imaging-based variables at both segmental and global scales, to derive four statistically stable clusters in SPIROMICS current smokers with unique structural and functional characteristics, and establish their associations with clinical metrics. Cluster 1 comprised relatively resistant smokers with preserved pulmonary function (FEV1/FVC > 0.7) and respiratory symptomatology (CAT> 10). Cluster 2 was characterized by airway wall thickening, fSAD-dominance, obesity and activity limitation. Cluster 3 exhibited airway wall thinning (in agreement with the findings of Smith et al. [36] and fSAD-dominance. Both Clusters 2 and 3 had FEV1/FVC close to the lower limit of normal, 0.7. Cluster 4 had mixed emphysema-fSAD with severe airway luminal narrowing, wall thinning and decreased lung function.

**Fig. 6:**
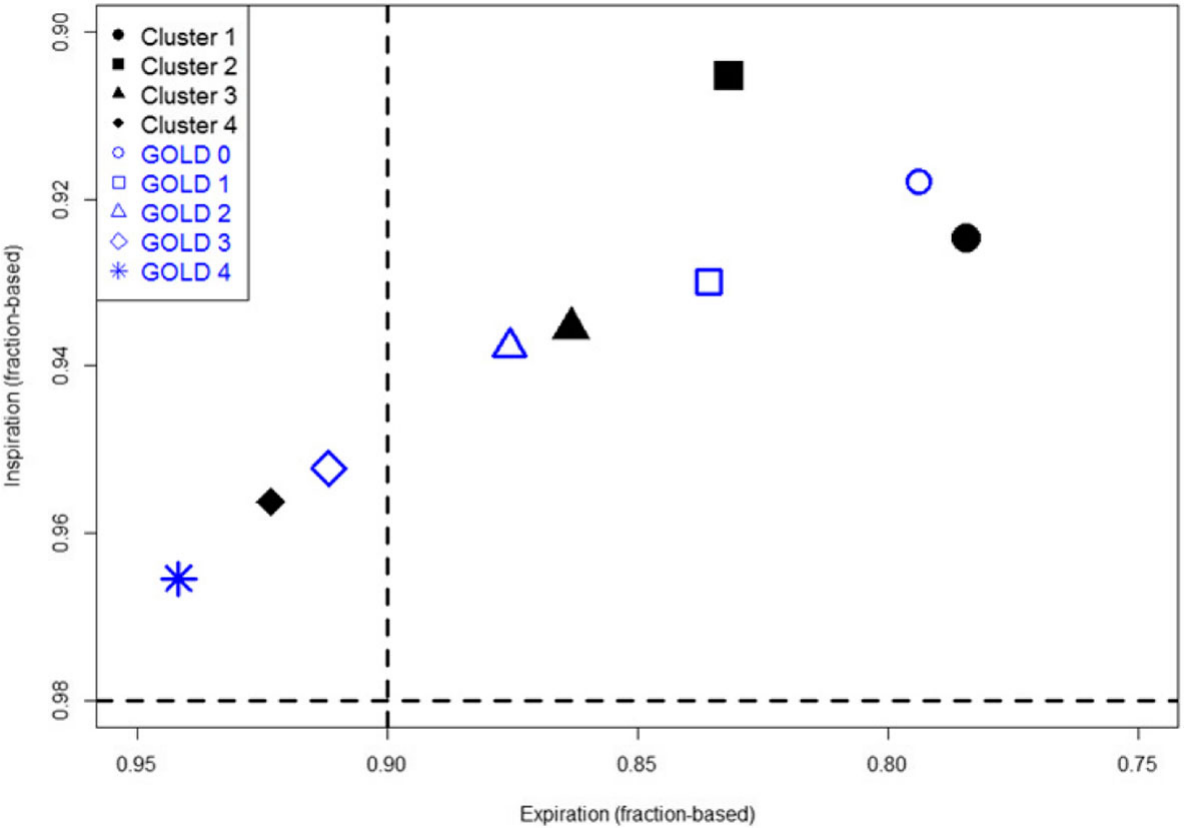
PRM based on GOLD stages and imaging-based derived clusters

**Fig. 7:**
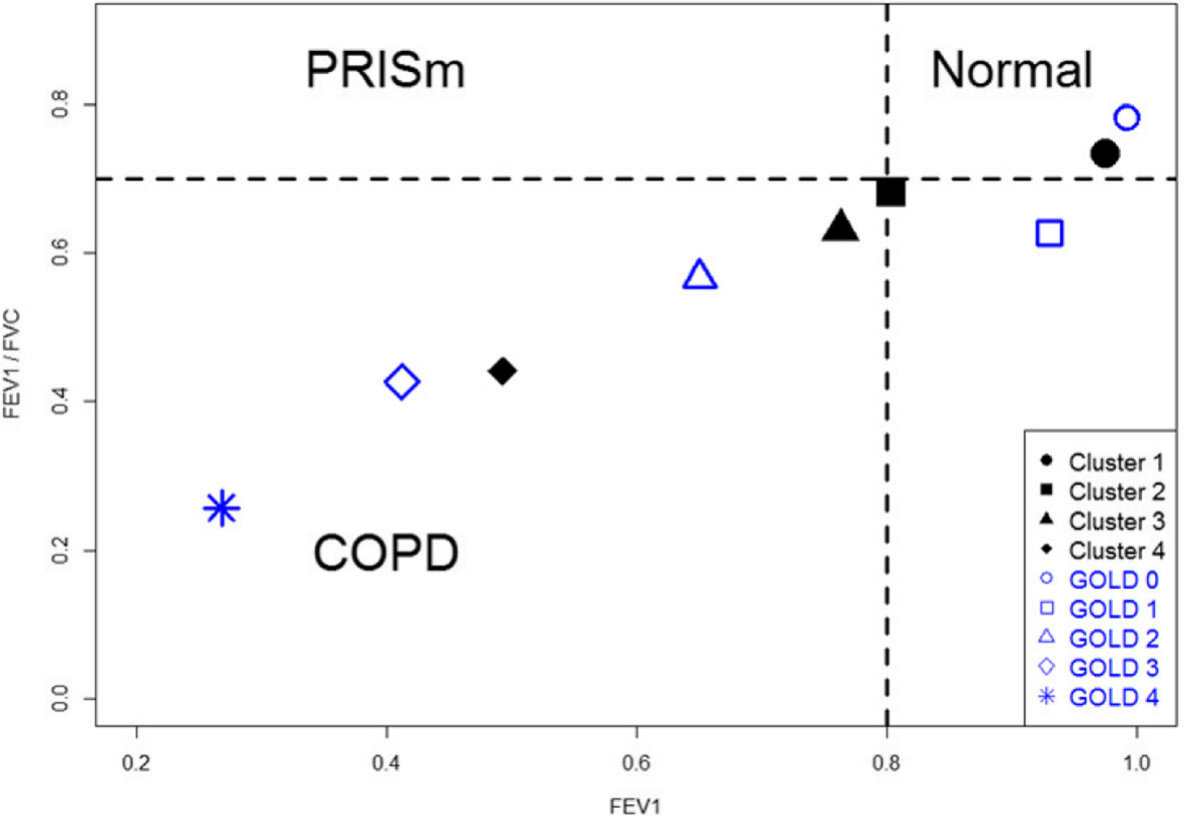
FEV1 and FEV1/FVC based of GOLD stages and imaging-based clusters. Dashed lines represent fixed threshold criteria (FEV1 = 0.8, FEV1/FVC = 0.7) used to distinguish possible PRISm subjects

To better understand the differences between spirometry-based GOLD stages and imaging-based clusters, Fig. 6 shows the distributions of GOLD 0–4 stages and Clusters 1–4 of the current smokers on a parametric response map (PRM) [28]. Except Cluster 2, Clusters 1, 3 and 4 appear to align with the path of the five GOLD stages. Wan et al. [37] studied a cohort of GOPDGene subjects with post-bronchodilator preserved ratio impaired spirometry (PRISm), characterized by a reduced FEV1 (< 0.8) with a preserved FEV/FVC ratio (≥0.7). They reported that PRISm subjects exhibit increased BMI, reduced 6-min walk, increased segmental airway wall area percentage, and increased respiratory symptoms [37], resembling both imaging and clinical characteristics of our Cluster 2. Thus, although only ~ 3% of the current smokers in this study met the spirometry criteria for PRISm, Fig. 7 displays the distributions of GOLD 0–4 stages and Clusters 1–4 of the same subjects on a post-bronchodilator FEV1-FEV1/FVC map. Cluster 2 is located nearest to the PRISm quadrant defined by the above spirometry criteria, as compared to GOLD 1 and 2. While a further study on a large PRISm cohort is needed to establish the link between imaging-based Cluster 2 and PRISm, the above analysis suggests that the current approach may be able to identify a clinically meaningful sub-population with COPD as compared with spirometric classification.

Castaldi et al. [14] classified four clusters in current and former smokers from the COPDGene study using four variables (features); FEV1% predicted, CT-quantified emphysema, segmental wall area% and emphysema distribution. Their four clusters are: relatively resistant smokers (i.e., no/mild obstruction and minimal emphysema despite heavy smoking), mild upper zone emphysema-predominant, airway disease-predominant and severe emphysema, with Clusters 2 and 4 having strong genetic associations. They included a PFT measure (FEV1% predicted) as one of the input features for cluster analysis, which is different from our MICA approach employing solely imaging-based variables to identify clusters and then establish associations of derived clusters with PFTs and other clinical measures and symptoms. Their clusters appear to overlap with our clusters. For example, our Cluster 1 (or 4) is similar to their Cluster 1 (or 4). Although our Cluster 2 and 3 had relatively lower FEV1/FVC (but being close to the cut-off threshold of 0.7) than that of Cluster 1, they exhibited a significantly increased fSAD% (*P* < 0.05) compared to Cluster 1 without a significant increase in Emph%. Thus, our Cluster 2, which exhibited increased fSAD%, thicker airway walls, the highest BMI, high BODE index and low Emph%, may correspond to their Cluster 3 being described as airway-predominant disease, thicker airway walls, lowest average emphysema of all clusters and high BMI. In addition, our Cluster 3 showed a relatively higher upper/lower emphysema ratio than others (Table 6); being similar to their Cluster 2 characterized by mild upper zone-predominant emphysema. Castaldi et al. [17] further investigated reproducibility of clustering analysis across multiple COPD cohorts using a set of common variables, suggesting that COPD heterogeneity may be characterized as a continuous trait.

**Table 6:** Upper/lower zone Emph% and fSAD%

Variable	Cluster 1 (*N* = 96)	Cluster 2 (*N* = 45)	Cluster 3 (*N* = 88)	Cluster 4 (*N* = 55)	*P* value
fSAD (U/L ratio)	9.54 (14.04)	5.1 (6.06)	5.92 (6.64)	1.63 (1.42)	< 0.0001
Emph (U/L ratio)	2.14 (3.15)	2.21 (2.36)	2.52 (3.42)	1.6 (1.85)	0.389
fSAD/Emph (% Total)	1.75 (1.67)	5.91 (7.44)	5.15 (4.16)	4.60 (4.72)	< 0.0001

Woodruff et al. [35] divided subjects (including both current and former smokers) from the SPIROMCS study into five categories A-E: (A) never smoked, preserved pulmonary function (B) CAT ≤10 (asymptotic); (C) CAT ≥10 (symptomatic), mild-to-moderate (GOLD stage 1 or 2); (D) CAT ≤10 and (E) CAT ≥10. The symptomatic subjects with preserved pulmonary function in category C had greater airway-wall thickness, but did not have higher Emph%, as compared with asymptotic subjects. These category-C subjects were younger with higher BMI and were more likely current smokers. These characteristics are strikingly similar to those of our Clusters 1 and 2 subjects. Cluster 1 included subjects that had thicker airway walls compared to Clusters 3 and 4, and had minimal-to-no emphysema. In addition, Cluster 2 exhibited several characteristics similar to Cluster 1, including lower symptomatology with CAT ≥10, thicker airway walls, minimal-to-no emphysema and FEV1/FVC = 0.68 (close to 0.74 for Cluster 1) as well as the highest BMI and β_tissue_ among all clusters. Nonetheless, different from Cluster 1 but similar to Cluster 4, Cluster 2 exhibited severe activity limitations and had relatively higher fSAD% and lower Jacobian. The major difference between Clusters 2 and 4 is that Cluster 2 had the highest BMI and β_tissue_. This suggests that symptomatic current smoker subjects in category C with preserved pulmonary function may be further divided into two sub-groups (Clusters 1 and 2) with distinct characteristics.

Garcia-Aymerich et al. [10] identified three groups in a cohort of 342 subjects recruited for the Phenotype and Course of COPD (PAC-COPD) study in Spain, using a comprehensive set of clinical, functional, biological and imaging metrics. Groups 1, 2 and 3 had respective FEV1/FVC of 0.44, 0.57 and 0.61. In addition to milder airflow limitation, Group 3 exhibited high BMI (obesity), systemic inflammation, cardiovascular disease, diabetes and activity limitation. These characteristics appear to overlap with those of our Cluster 2. While both Clusters 2 and 3 were fSAD-dominant subjects, they were characterized by increased and decreased airway wall-thickness, respectively. Also, Sood et al. [38] suggested that higher BMI (obesity) might contribute to systemic inflammation.

Our study here has several limitations. It focused on current smokers and was a cross-sectional study. In the future, the analysis shall be extended to include former smokers and compared with the current analysis. Also, our analysis will be extended to longitudinal data and cross validation shall be performed to examine cluster transition and stability over time. As a preliminary study, we included in the Additional file 1 the cluster analysis of longitudinal data from a small COPD cohort, showing consistently four stable clusters. While our use of image matching is refined to the level of accounting for lobar slippage, it requires segmentation of the lobes at both inspiration and expiration.

## Conclusions

In conclusion, using a *K-means* clustering method we found four distinct stable clusters of COPD subtypes. These are Cluster 1, non-severe COPD with normal airway structure (relatively resistant smoker); Cluster 2, a mix of non-severe and severe COPD with fSAD dominance, low emphysema percentage, high tissue fraction with wall thickening; Cluster 3, a mix of non-severe and severe COPD, fSAD dominance with decreased wall thickness and luminal narrowing; Cluster 4, a mix of severe fSAD and emphysema with significant alterations in functional and structural variables. A decision tree analysis with only 7 discriminant imaging-based variables allows classification with an accuracy close to the “original” cluster membership. The unique structural and functional characteristics observed in each cluster can help shed light on the existing heterogeneous nature of the disease.

## Additional file

Additional file 1: **Table S1.** Standardized loadings of seven principal components based upon correlation matrix. **Table S2.** Major structural and functional imaging-based variables in four imaging-based clusters for 45 current smokers from longitudinal study. **Figure S1.** Clustering analysis, a: Internal property in different clustering methods; b: Clustering stability analysis between K-means and Hierarchical clustering with different number of clusters. **Figure S2.** Cluster analysis in training set (a) and validation set (b) with four clusters. **Figure S3.** A scree plot for determining the optimal number of principal components for longitudinal study. (DOCX 276 kb)

## Abbreviations

ADI: Anisotropic deformation index
BMI: Body mass index
BronInt: Right intermediate bronchus
*Cr*: Airway luminal circularity
*D*_h_: Hydraulic luminal diameter
Emph%: Emphysema percentage
FEV_1_: Forced expiratory volume in one second
fSAD%: Functional small airway disease percentage
Jacobian: Determinant of Jacobian matrix
LA: Airway luminal area
LLL: Left lower lobe
LMB: Left main bronchus
LUL: Left upper lobe
Lung shape: Apical-basal distance over ventral-dorsal distance at TLC
MICA: Multiscale imaging-based cluster analysis
PCA: Principal component analysis
PFT: Pulmonary function test
PRISm: Preserved ratio impaired spirometry
QCT: Quantitative computed tomography
RLL: Right lower lobe
RMB: Right main bronchus
RML: Right middle lobe
RUL: Right upper lobe
RV: Residual volume
sLLL: Sub-grouped left lower lobe with branches of LB6, LB8
sLUL: Sub-grouped left upper lobe with branches of LB1
SPIROMICS: Subpopulations and intermediate outcome measures in COPD study
sRLL: Sub-grouped right lower lobe with branches of RB6
sRML: Sub-grouped right middle lobe with branches of RB4
sRUL: Sub-grouped right upper lobe with branches of RB1
TLC: Total lung capacity
TriLLB: Trifurcation of left lower lobe
U/(M + L)|v: The ratio of air volume changes in upper lobes to those in middle and lower lobes
WA%: Airway wall area percentage, i.e., the ratio of wall area to total area
WBC: White blood cell
WT: Airway wall thickness
β_tissue_: Tissue fraction
Δ*V*_air_^F^: Lobar fraction of air volume change between TLC and RV
*θ*: Bifurcation angle between two daughter branches
