# Supplementary material for: Imaging-based clusters in current smokers of the COPD cohort associate with clinical characteristics: the SubPopulations and Intermediate Outcome Measures in COPD Study (SPIROMICS)
Source: Respir Res. 2018 Sep 18;19:178. doi: 10.1186/s12931-018-0888-7 (PMC6145340; doi:10.1186/s12931-018-0888-7)
Supplement: Supplementary file 1 — Table S1. Standardized loadings of seven principal components based upon correlation matrix. Table S2. Major structural and functional imaging-based variables in four imaging-based clusters for 45 current smokers from longitudinal study. Figure S1. Clustering analysis, a: Internal property in different clustering methods; b: Clustering stability analysis between K-means and Hierarchical clustering with different number of clusters. Figure S2. Cluster analysis in training set (a) and validation set (b) with four clusters. Figure S3. A scree plot for determining the optimal number of principal components for longitudinal study. (DOCX 276 kb) [file 12931_2018_888_MOESM1_ESM.docx]

# SUPPLEMENTARY RESULTS

## Principal Component Analysis

With 69 imaging variables, the data may be too large to study and interpret properly. Also there would be many pairwise correlation between variables. A principal component analysis (PCA) was performed to derive linearly uncorrelated variables (principal components) from a set of correlated variables (E1–E4) while capturing as much information in the original variables as possible. The lower dimension principal components were then used to feed clustering analysis.

As such, the optimal number of principal components was determined as seven explaining 64% of the variation in the original data (Table E1). Seven components loadings (the correlations of the imaging variables with the principal components) were tabulated in Table E1.The first component was related to β_tissue_, fSAD% and Emph%; The second component was related to Jacobian and ADI; The third component was related WT*, shape at TLC and *Cr*; The fourth component was related U/(M+L)|v, ΔV_air_^F^ and *θ* (RMB); The fifth component was related *D*_h_* and ΔV_air_^F^ (RML); The sixth component was related *D*_h_* and WT* (RMB); The seventh component was related to *Cr*.

Table E1. Standardized loadings of seven principal components based upon correlation matrix. The variables with the significant correlation in each component are indicated by an *asterisk*. Full names of each variable were described in *Abbreviations used* section of the main text. These 69 variables consist of 32 local (*θ* in two regions, *Cr*, WT* and *D*_h_* in ten regions), 30 lobar (Δ*V*_air_^F^, fSAD%, Emph%, β_tissue_%, Jacobian and ADI in five lobes) and 7 global (lung shape, U/(M+L)|v, fSAD%, Emph%, β_tissue_% , Jacobian and ADI) variables.

|  | | | Principal components | | | | | | |
| --- | --- | --- | --- | --- | --- | --- | --- | --- | --- |
|  |  |  | 1 | 2 | 3 | 4 | 5 | 6 | 7 |
| Proportion of variation | | | 25 | 11 | 8.8 | 6.8 | 5.3 | 3.5 | 3 |
| Cumulative  Variation | | | 25 | 37 | 45.8 | 52.5 | 57.8 | 61.3 | 64.3 |
|  | | | | | | | | |  |
| Variable | Region | | Standardized loadings based upon correlation matrix | | | | | | |
| *θ* | Trachea | | 0.14 | 0.3 | -0.04 | 0.38 | -0.29 | -0.09 | -0.11 |
|  | RMB | | -0.32 | -0.28 | 0.17 | -0.13 | -0.15 | -0.28 | -0.1 |
| *Cr* | Trachea | | 0.21 | -0.17 | 0.22 | -0.25 | 0.35 | 0.13 | 0.35 |
|  | RMB | | 0.35 | -0.31 | 0.15 | -0.42 | 0.22 | 0.21 | 0.31 |
|  | LMB | | 0.4 | -0.21 | 0.19 | -0.23 | 0.02 | 0.29 | 0.28 |
|  | TriLLB | | 0.12 | -0.18 | 0.06 | -0.12 | -0.13 | 0.08 | 0.42 |
|  | BronInt | | 0.39 | -0.06 | 0.01 | -0.07 | -0.04 | 0.47 | 0.47 |
|  | sLUL | | 0.16 | -0.13 | -0.02 | -0.15 | -0.12 | 0.13 | 0.14 |
|  | sLLL | | 0.01 | -0.21 | 0.02 | -0.14 | -0.27 | 0.34 | 0.1 |
|  | sRUL | | 0.07 | -0.08 | 0 | -0.04 | -0.02 | 0.16 | 0.33 |
|  | sRML | | 0.06 | -0.03 | -0.03 | 0.09 | -0.11 | 0.08 | 0.01 |
|  | sRLL | | 0 | -0.19 | 0.05 | -0.05 | -0.06 | 0.09 | 0.2 |
| WT* | Trachea | | 0.06 | -0.03 | 0.1 | 0.46 | -0.29 | 0.22 | -0.17 |
|  | RMB | | 0.28 | -0.05 | 0.24 | 0 | 0.23 | 0.18 | -0.33 |
|  | LMB | | 0.2 | -0.12 | 0.36 | 0.13 | 0.27 | 0.3 | -0.32 |
|  | TriLLB | | 0.31 | -0.04 | 0.34 | 0.55 | -0.28 | 0.12 | 0.06 |
|  | BronInt | | 0.21 | -0.02 | 0.25 | 0.46 | -0.15 | 0.41 | 0.1 |
|  | sLUL | | 0.41 | 0.03 | 0.35 | 0.59 | -0.11 | -0.09 | 0.11 |
|  | sLLL | | 0.39 | 0 | 0.32 | 0.6 | -0.31 | 0.05 | 0.12 |
|  | sRUL | | 0.3 | 0.14 | 0.26 | 0.64 | -0.22 | 0.07 | 0.11 |
|  | sRML | | 0.38 | 0.09 | 0.3 | 0.56 | -0.15 | -0.07 | -0.02 |
|  | sRLL | | 0.39 | 0 | 0.36 | 0.57 | -0.26 | -0.06 | 0.09 |
| *D*_h_* | Trachea | | 0.19 | -0.22 | 0.52 | -0.14 | 0.44 | 0.21 | -0.1 |
|  | RMB | | 0.44 | -0.11 | 0.46 | -0.09 | 0.45 | 0.3 | -0.13 |
|  | LMB | | 0.37 | -0.2 | 0.45 | 0 | 0.36 | 0.32 | -0.27 |
|  | TriLLB | | 0.57 | -0.04 | 0.52 | -0.05 | 0.31 | -0.21 | 0.01 |
|  | BronInt | | 0.47 | -0.08 | 0.38 | 0.04 | 0.21 | 0.43 | -0.02 |
|  | sLUL | | 0.53 | -0.09 | 0.39 | 0.18 | 0.36 | -0.33 | 0.03 |
|  | sLLL | | 0.57 | -0.1 | 0.51 | 0.09 | 0.21 | -0.31 | 0.08 |
|  | sRUL | | 0.51 | -0.05 | 0.4 | 0.19 | 0.35 | -0.24 | 0.07 |
|  | sRML | | 0.49 | -0.04 | 0.4 | 0.12 | 0.29 | -0.28 | -0.11 |
|  | sRLL | | 0.56 | -0.09 | 0.5 | 0.08 | 0.22 | -0.34 | 0.02 |
| Shape at TLC | | | -0.14 | -0.26 | 0.15 | -0.31 | 0.27 | -0.09 | -0.05 |
| U/(M+L)\|v | | | 0.06 | 0.45 | -0.53 | 0.3 | 0.54 | 0.09 | -0.03 |
| ΔV_air_^F^ | | LUL | 0.16 | 0.36 | -0.5 | 0.31 | 0.51 | 0.04 | 0.03 |
|  |  | LLL | -0.22 | -0.38 | 0.48 | -0.28 | -0.47 | -0.04 | 0.13 |
|  |  | RUL | 0.05 | 0.34 | -0.54 | 0.3 | 0.48 | 0.19 | 0.01 |
|  |  | RML | 0.14 | 0.09 | -0.19 | 0.15 | -0.11 | 0.12 | -0.19 |
|  |  | RLL | -0.03 | -0.29 | 0.56 | -0.33 | -0.38 | -0.23 | -0.07 |
| β_tissue_ | | Total | 0.57 | 0.75 | -0.03 | -0.06 | -0.06 | -0.07 | 0.1 |
|  |  | LUL | 0.53 | 0.73 | 0 | -0.04 | -0.1 | -0.05 | 0.08 |
|  |  | LLL | 0.5 | 0.76 | -0.08 | -0.04 | 0.08 | -0.17 | 0.16 |
|  |  | RUL | 0.55 | 0.66 | 0.05 | -0.04 | -0.22 | 0.06 | -0.06 |
|  |  | RML | 0.51 | 0.66 | 0.03 | -0.09 | 0.03 | -0.13 | 0.15 |
|  |  | RLL | 0.54 | 0.75 | -0.12 | -0.03 | 0.05 | -0.09 | 0.15 |
| Emph% | | Total | -0.68 | -0.34 | -0.08 | 0.32 | 0.25 | -0.15 | 0.31 |
|  |  | LUL | -0.6 | -0.35 | -0.09 | 0.29 | 0.24 | -0.15 | 0.3 |
|  |  | LLL | -0.63 | -0.34 | 0 | 0.29 | 0.09 | 0.03 | 0.09 |
|  |  | RUL | -0.52 | -0.26 | -0.1 | 0.23 | 0.31 | -0.25 | 0.4 |
|  |  | RML | -0.57 | -0.24 | -0.16 | 0.33 | 0.07 | -0.02 | 0.23 |
|  |  | RLL | -0.69 | -0.3 | 0 | 0.3 | 0.12 | -0.07 | 0.17 |
| fSAD% | | Total | -0.9 | -0.13 | 0.11 | 0.2 | 0.09 | 0.04 | -0.09 |
|  |  | LUL | -0.88 | -0.16 | 0.13 | 0.11 | 0.03 | 0.02 | -0.09 |
|  |  | LLL | -0.78 | -0.11 | 0.08 | 0.29 | 0.08 | 0.15 | -0.14 |
|  |  | RUL | -0.84 | -0.16 | 0.12 | 0.1 | 0.11 | -0.08 | 0 |
|  |  | RML | -0.81 | -0.12 | 0.11 | 0.12 | -0.03 | 0.05 | -0.15 |
|  |  | RLL | -0.81 | -0.09 | 0.11 | 0.24 | 0.11 | 0.1 | -0.07 |
| Jacobian | | Total | 0.76 | -0.52 | -0.27 | 0 | -0.07 | -0.01 | 0.01 |
|  |  | LUL | 0.73 | -0.41 | -0.34 | 0.09 | 0 | 0.02 | 0.04 |
|  |  | LLL | 0.7 | -0.58 | -0.14 | -0.1 | -0.1 | -0.09 | 0.04 |
|  |  | RUL | 0.73 | -0.42 | -0.36 | 0.09 | -0.02 | 0.08 | 0 |
|  |  | RML | 0.67 | -0.46 | -0.3 | 0.07 | -0.08 | 0.08 | -0.03 |
|  |  | RLL | 0.71 | -0.6 | -0.14 | -0.09 | -0.1 | -0.09 | -0.03 |
| ADI | | Total | 0.65 | -0.53 | -0.41 | 0.13 | 0 | -0.1 | -0.11 |
|  |  | LUL | 0.62 | -0.37 | -0.49 | 0.25 | 0.05 | -0.02 | -0.06 |
|  |  | LLL | 0.53 | -0.54 | -0.31 | 0.03 | -0.01 | -0.2 | -0.03 |
|  |  | RUL | 0.6 | -0.33 | -0.5 | 0.25 | 0.08 | 0.07 | -0.08 |
|  |  | RML | 0.49 | -0.57 | -0.29 | 0.04 | -0.1 | -0.02 | -0.14 |
|  |  | RLL | 0.55 | -0.52 | -0.26 | 0.04 | 0.01 | -0.22 | -0.15 |

## Clustering Analysis

For a given complex data set such as imaging variables, choosing the appropriate clustering method and then finding the optimal number of clusters are important. We used the package *clValid* in R software to assess internal validation measures of clustering. The internal measures included in *clValid* are *Connectivity*, *Average Silhouette width* and *Dunn index*.

*Connectivity* indicates the degree of connectedness of the clusters, as determined by k-nearest neighbors. Connectedness relates to what extent items are placed in the same cluster as their nearest neighbors. The connectivity has a value between 0 and infinity and should be minimized.

The *Dunn index* is the ratio between the smallest distance between observations not in the same cluster to the largest intra-cluster distance. It has a value between 0 and infinity and should be maximized. *Silhouette width* corresponds to the degree of confidence in particular clustering assignment. Smaller *connectivity* and larger *Silhouette* *width* and *Dunn index* indicate the better quality of clustering. *Connectivity* criteria suggested *K-means* and hierarchical methods are good choices for the current imaging data based (Figure E1a).

To find the optimal number of clusters, a cluster stability analysis was performed with a non-parametric bootstrap analysis, based on resampling from the raw imaging data (E5) for different number of clusters and then the mean of Jaccard similarity coefficients was calculated to compare the cluster patterns derived from the bootstrapped datasets with the original clusters.

*K-means* clustering outperformed the Hierarchical clustering with more stable results (Figure E1b). *K-means* method with 4 clusters reached and remained in the more stable region (E > 0.85). A package “NbClust” in software *R* (E6) was used to perform stability analysis.

| 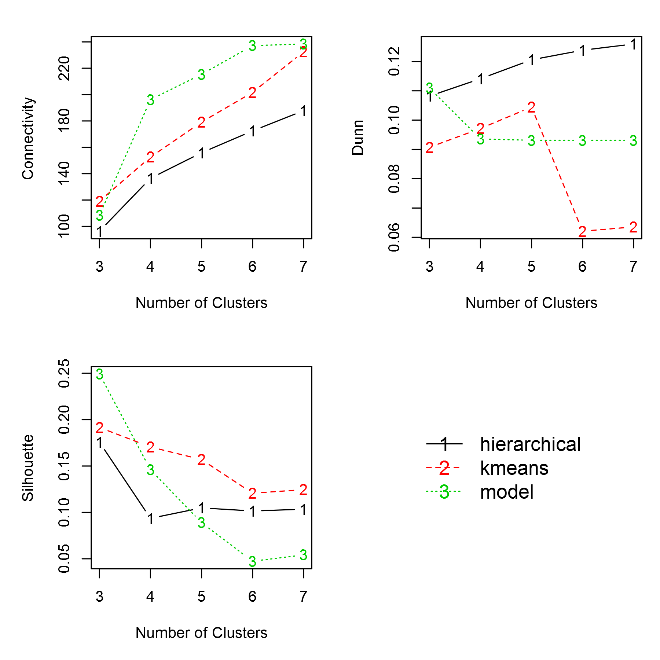 | 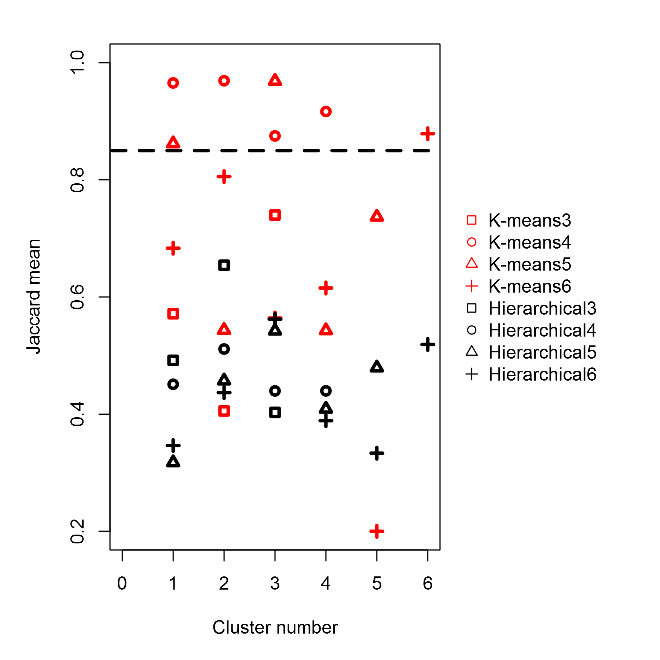 |
| --- | --- |
| a. Internal property | b. Clustering stability |
| Fig. E1: Clustering analysis, a: Internal property in different clustering methods; b: Clustering stability analysis between *K-means* and Hierarchical clustering with different number of clusters. | |

## Validation of Cluster Characteristics

After choosing the stable cluster solution with the meaningful correlation with the clinical measures, the repeatability (validation) of the cluster analysis can be assessed by dividing the data set into training and validation (testing) sets. First, the data set was shuffled randomly into training (n = 227) and test sets (n = 57) with the 80/20 ratio, respectively. Then clusters were assigned in the validation set by assigning each subject to the closest cluster centers learned by the *K-means* algorithm in the training set (Figures E2 (a) and (b)). T-test showed no significant difference for cluster characteristics between training and validation sets (*P* > 0.05), meaning the repeatability of the cluster solution.

| 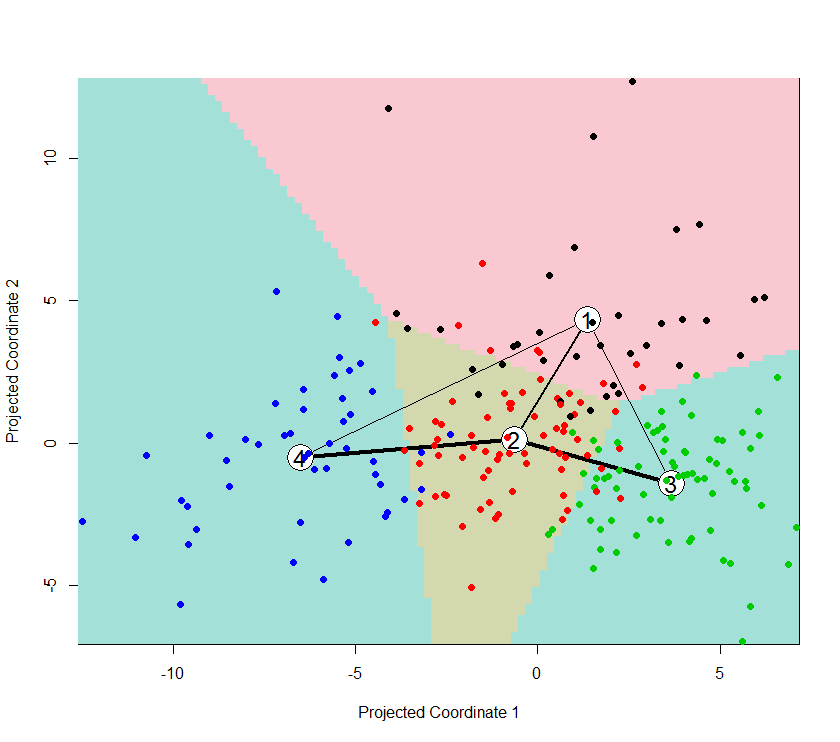 | 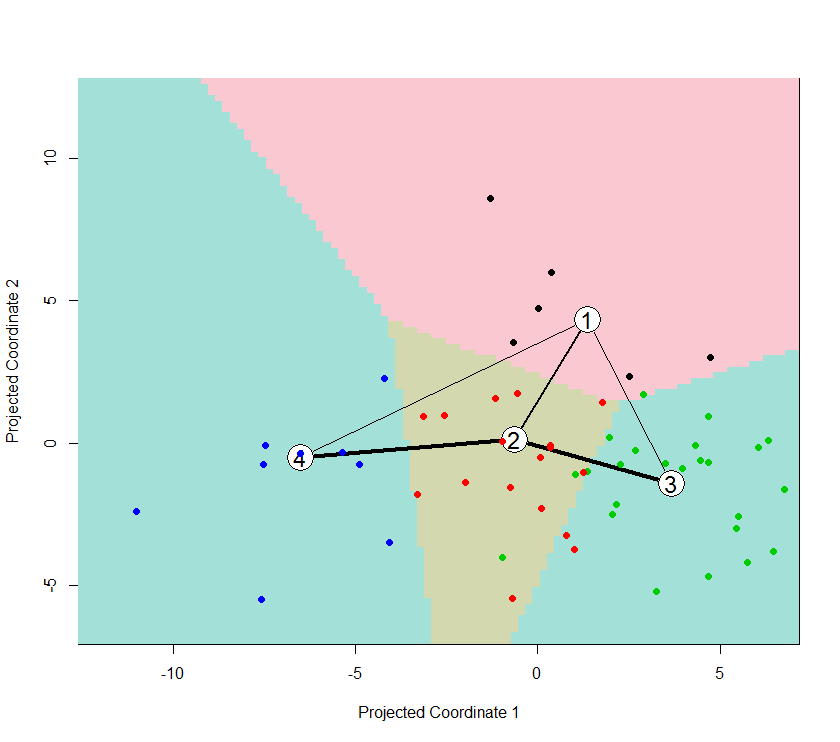 |
| --- | --- |
| (a) | (b) |
| Fig. E2: Cluster analysis in training set (a) and validation set (b) with four clusters. | |

## Longitudinal Study

As a preliminary study, 123 subjects recruited into SPIROMICS longitudinal study were assessed. After segmentation, image matching from TLC to RV was performed. From these subjects with matching data, we analyzed 45 current smokers (N = 45). Using extracted imaging-based features, principal component analysis (Figure E3) and cluster analysis (Table E2) were performed. The K-means clustering method produced four stable and unique clusters, containing 21, 6, 5 and 13 subjects, respectively, with a set of major variables being consistent with those of the baseline analysis (Table E2). Longitudinal data can be used to track the disease alteration at multiscale levels for better assessment of the disease.

| 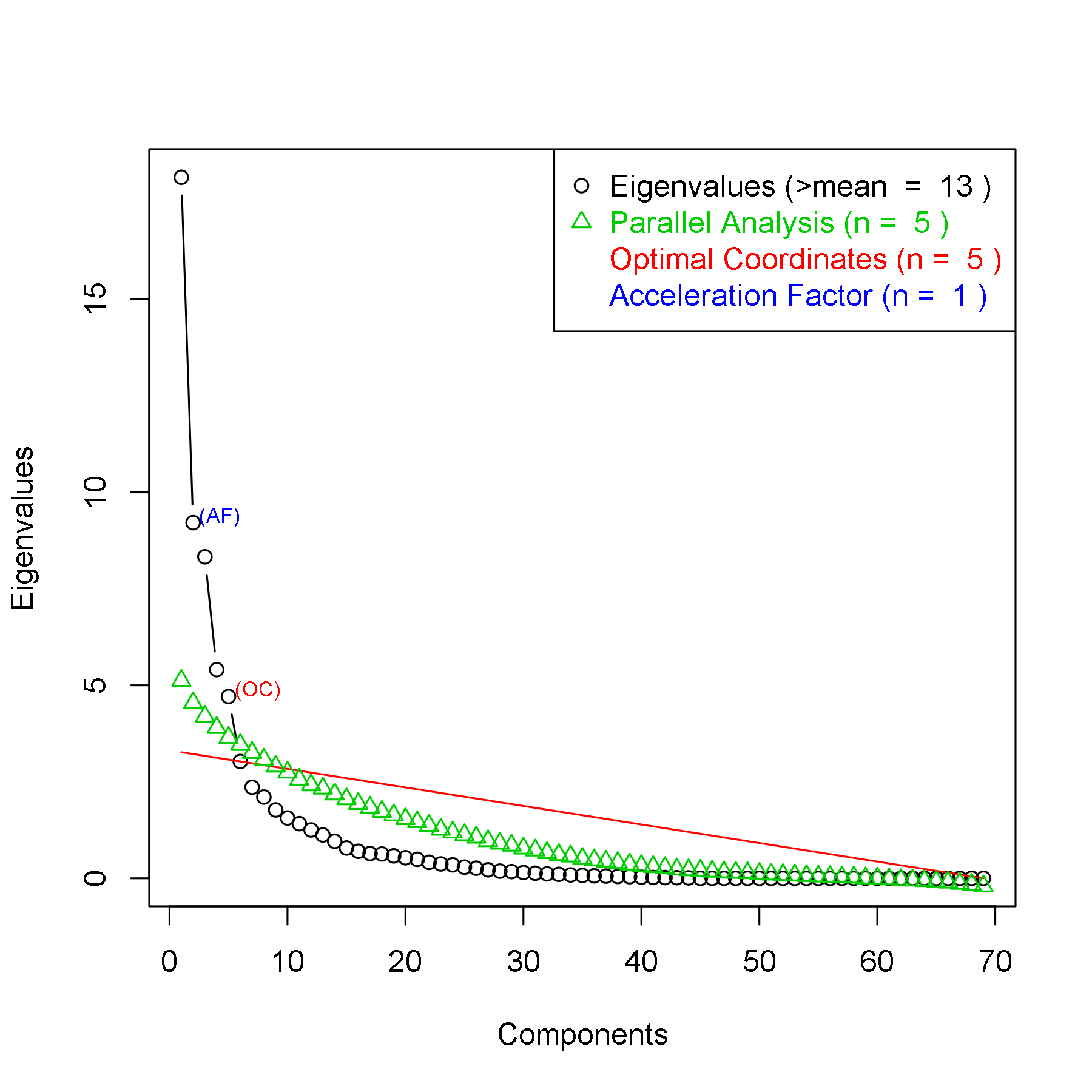 |
| --- |
| Fig. E3: A scree plot for determining the optimal number of principal components for longitudinal study. |

Table E2. Major structural and functional imaging-based variables in four imaging-based clusters for 45 current smokers from longitudinal study.

| Variable | Region | Cluster 1  (N = 21) | Cluster 2  (N = 6) | Cluster 3  (N = 5) | Cluster 4  (N = 13) | *P* value |
| --- | --- | --- | --- | --- | --- | --- |
| fSAD% | Total | 5.3 | 1.9 | 6.8 | 34.5 | <0.0001 |
| Jacobian | Total | 2.1 | 1.4 | 1.5 | 1.4 | <0.0001 |
| β_tissue_ | Total | 0.11 | 0.13 | 0.15 | 0.098 | <0.0001 |
| WT^*^ | sRML | 0.59 | 0.63 | 0.56 | 0.55 | <0.0001 |
| ADI | RUL | 0.39 | 0.21 | 0.33 | 0.22 | <0.0001 |
| *D*_h_* | sLLL | 0.35 | 0.37 | 0.29 | 0.3 | <0.0001 |
| Emph% | Total | 2.5 | 1.5 | 1.1 | 8.5 | <0.0001 |
| ADI | Total | 0.48 | 0.26 | 0.35 | 0.24 | <0.0001 |
| Δ*V*_air_^F^ | LLL | 0.26 | 0.32 | 0.17 | 0.25 | <0.0001 |
| Cr | LMB | 0.98 | 0.96 | 0.96 | 0.94 | <0.0001 |

**REFERENCES**

E1. Hayton JC, Allen DG, Scarpello V. Factor Retention Decisions in Exploratory Factor Analysis: a Tutorial on Parallel Analysis. *Organ Res Methods* 2004;7:191–205.

E2. Weatherall M, Shirtcliffe P, Travers J, Beasley R. Use of cluster analysis to define COPD phenotypes. *Eur Respir J* 2010;36:472–474.

E3. Paoletti M, Camiciottoli G, Meoni E, Bigazzi F, Cestelli L, Pistolesi M, Marchesi C. Explorative data analysis techniques and unsupervised clustering methods to support clinical assessment of Chronic Obstructive Pulmonary Disease (COPD) phenotypes. *J Biomed Inform* 2009;42:1013–1021.

E4. Johnson & Wichern, Applied Multivariate Statistical Analysis, 6th Edition. at <https://www.pearsonhighered.com/program/Johnson-Applied-Multivariate-Statistical-Analysis-6th-Edition/PGM274834.html>.

E5. Hennig C. *fpc: Flexible Procedures for Clustering*. 2015. at <https://cran.r-project.org/web/packages/fpc/index.html>.

E6. NbClust function | R Documentation. at <https://www.rdocumentation.org/packages/NbClust/versions/3.0/topics/NbClust>.
